# Evaluation of Smartphones Equipped with Light Detection and Ranging Technology for Circumferential and Volumetric Measurements in Lower Extremity Lymphedema

**DOI:** 10.3390/bios15060381

**Published:** 2025-06-12

**Authors:** Masato Tsuchiya, Kanako Abe, Satoshi Kubo, Ryuichi Azuma

**Affiliations:** 1 Department of Plastic and Reconstructive Surgery, National Defense Medical College, Saitama 3598513, Japan; mashi-1984@live.jp (S.K.); azuma@ndmc.ac.jp (R.A.); 2 Center for Palliative Care, National Defense Medical College, Saitama 3598513, Japan; jancj53148@yahoo.co.jp

**Keywords:** lymphedema, LiDAR, smartphones, circumference measurement, volumetric measurement

## Abstract

Lower extremity lymphedema (LEL) requires precise limb measurements for treatment evaluation and compression garment design. Tape measurement (TM) is the standard method but is time-consuming. Smartphones with light detection and ranging (LiDAR) technology may offer fast and efficient alternatives for three-dimensional imaging and measurement. This study evaluated the accuracy, reliability, and time efficiency of LiDAR measurements compared with those of TM in patients with LEL. A healthy volunteer and 55 patients were included. Circumferences of the foot, ankle, calf, knee, and thigh and the volume were measured using TM and smartphones with LiDAR. The water displacement method was used to validate volume measurements. The measurement time, reliability, correlation, agreement, and systematic differences between the methods were assessed. LiDAR showed excellent reliability in the healthy volunteer (inter-rater intraclass correlation coefficients: 0.960–0.988) and significantly reduced the measurement time compared with TM (64.0 ± 15.1 vs. 115.3 ± 30.6 s). In patients with LEL, strong correlations and agreements were observed for ankle, calf, and knee measurements. However, foot and thigh measurements showed lower correlations and larger discrepancies. LiDAR has excellent accuracy and reliability in measuring the circumference and volume of the lower leg and has the potential to reduce the time required to acquire data. Limitations include lower accuracy for foot and thigh measurements and the current workflow complexity, which requires the use of multiple software tools.

## 1. Introduction

Lower extremity lymphedema (LEL) is a chronic disease characterized by the subcutaneous accumulation of protein-rich fluid due to lymphatic dysfunction in the lower limbs, causing cosmetic and functional impairment with significant physical and psychological morbidity. The most basic treatment for LEL involves wearing a compression garment. Circumferential measurements of the affected limbs using tape measurement (TM) are commonly used to evaluate the condition and effectiveness of the therapy in daily practice. Measurements are commonly made at the foot, ankle, calf, knee, and thigh. Moreover, to create a well-fitting compression garment, it is essential to measure the limb circumference using TM [1]. Several circumferential measurements are required to fabricate compression garments, and, occasionally, the measurement sites vary from manufacturer to manufacturer. Although TM is inexpensive, it is time-consuming and burdensome for patients and clinicians if there are several measurement sites.

Light detection and ranging (LiDAR) is an active remote sensing technology that uses laser pulses to generate accurate three-dimensional (3D) point clouds and digital elevation models. It operates by measuring scattered light to determine the range and other information regarding distant objects [2]. LiDAR is similar to a 3D laser scanner; however, it can measure a wide area while moving. This technology is widely used in autonomous vehicles for navigation and environment mapping and offers precise depth perception and 3D mapping capabilities [3,4]. Although LiDAR has the disadvantage of being expensive, a low-cost LiDAR that can be installed on smartphones has recently been introduced. The integration of a LiDAR scanner with a smartphone camera allows for the effortless capture of 3D images of various objects, providing accurate details of their form and surface [5].

Measurements using 3D images created using LiDAR have been widely used in various fields. However, few studies have demonstrated its effectiveness in the field of biometrics [5,6,7]. In this study, we evaluated the effectiveness of LiDAR measurements in patients with LEL by comparing LiDAR with TM. 

## 2. Materials and Methods

This study was conducted at the National Defense Medical College (Saitama, Japan), with approval from the Institutional Review Board and permission from the ethics committee (no. 5077).

### 2.1. Evaluation of the Validity and Reliability of LiDAR in a Healthy Volunteer

Three examiners, including one plastic surgeon, one expert nurse in lymphedema, and one medical student, measured the right leg of one healthy volunteer using TM (Smartbody Measure®, Renpho Inc., Anaheim, CA, USA) three times. All examiners had little to no experience in handling LiDAR devices.

**Figure 1:**
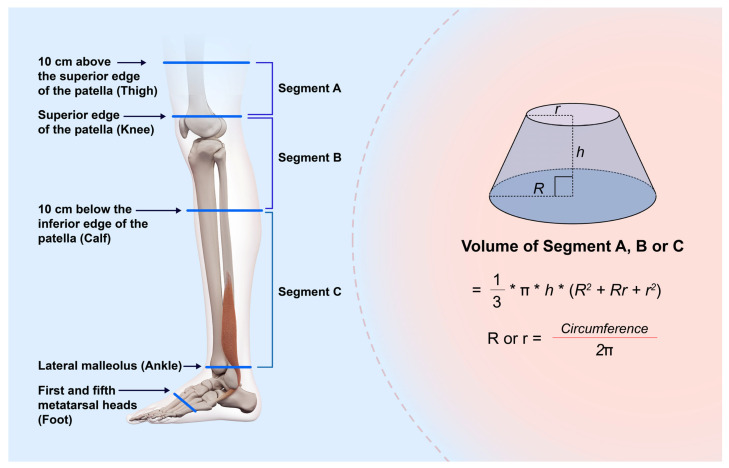
Schema at the time of measurement. The limb circumference is measured at the superior edge of the patella (knee), 10 cm above and below the patella (thigh and calf, respectively), the lateral malleolus (ankle), and the first and fifth metatarsal heads (foot) in the standing position. Vertical distances between the thigh and the knee (segment A), between the knee and the calf (segment B), and between the calf (segment C) and the ankle are measured using TM. Each segment is regarded as a circular truncated cone, and its volume is calculated. R and r: radii calculated based on the circumference, h: Height of the truncated cone.

The lower extremity circumference measurements of the volunteer were taken at the superior edge of the patella (knee), 10 cm above the superior edge of the patella (thigh), 10 cm below the inferior edge of the patella (calf), the lateral malleolus (ankle), and the first and fifth metatarsal heads (foot) in the standing position. Moreover, the vertical distances between the thigh and the knee (segment A), between the knee and the calf (segment B), and between the calf and the ankle were measured using TM. The time required for each measurement was recorded. The volumes in segments A, B, and C were estimated based on circumference measurements and vertical distances. Each segment was considered a truncated cone with a circular base, and its volume was determined (Figure 1). 

A 3D model was generated using a smartphone (iPhone 15 Pro®, Apple Inc., Cupertino, CA, USA) equipped with LiDAR by capturing the surface data of the right leg of the volunteer in the standing position. First, lines were drawn on the foot, ankle, calf, and thigh of the lower extremity to be captured. The device was gradually moved in a circular trajectory around the leg to collect surface data from multiple angles, with the LiDAR sensor oriented perpendicular to the limb surface to optimize data acquisition. The 3D Scanner App® (LaanLabs, New York, NY, USA), a free application, was used to acquire the data and generate the 3D images. In this application, the point cloud was configured to be acquired at approximately 5 mm intervals. According to the manufacturer, the LiDAR system is capable of capturing depth data at up to 30 frames per second [8]. The time required to obtain the 3D images was recorded. This process was performed three times by the three examiners.

**Figure 2:**
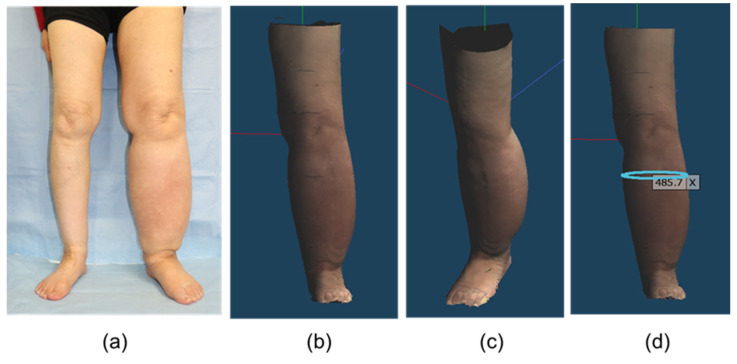
3D images in the patient with lymphedema captured by the smartphone equipped with LiDAR on Metasequoia 4^®^: (**a**) Clinical photograph (**b**) Front and (**c**) oblique views. The 3D image can be freely rotated in the software. (**d**) Measurement of the circumferential diameter in any cross section (unit: mm). LiDAR, light detection and ranging; 3D, three-dimensional.

Metasequoia 4® (Tetraface, Tokyo, Japan), a 3D computer graphics software for Windows® (Microsoft Corp., Redmond, WA, USA) and Mac® (Apple Inc., Cupertino, CA, USA) operating systems, was used to process the obtained 3D models. It has a specific tool for measuring the circumference and distance of interest directly on a 3D model. We measured the circumferences of the foot, ankle, calf, knee, and thigh of the volunteer using this tool. Blender® (Blender Foundation, Nood-Holland, The Netherlands), an open-source software for Windows® and Mac®, offers a specific tool for measuring the volume of a 3D model. The volumes of segments A, B, and C were measured using this tool (Figure 2). 

The water displacement method was used to accurately determine the volume of the volunteer’s leg. 

A glass container was filled with water, and the right lower limb was immersed in water up to the knee. When the limb was submerged, water flowed from the container into the secondary container. The displaced water was collected, and its volume was measured using a graduated cylinder. The volumes of the toe and ankle were measured using a similar technique, and the ankle volume was subtracted from the knee volume. This volume directly corresponded to the volumes of segments B and C.

### 2.2. Agreement and Correlation Between TM and LiDAR in Patients with LEL

Participants who were referred to our hospital to create a compression garment for LEL between January 2024 and February 2025 were initially included. Exclusion criteria were patients who could not stand by themselves, as TM or LiDAR measurements were conducted in the standing position. The following patient characteristics were collected: sex, age, cause of lymphedema, International Society of Lymphology classification, lymphedema duration, and body mass index. Both legs were measured using TM and LiDAR by a nurse with expertise in lymphedema, regardless of whether the limb was affected. TM and LiDAR measurements were performed once on one leg. TM were performed at the same sites as those in the volunteer. Methods to obtain 3D models using a smartphone equipped with LiDAR and to measure the circumferences of the foot, ankle, calf, knee, and thigh as well as the volume of segments A, B, and C on 3D models were also performed in the same manner as that in the volunteer. 

Consent to participate in this study was obtained from all participants. 

### 2.3. Statistical Analysis

Continuous data are expressed as means ± standard deviation (SD). The inter-rater reliability of circumference measurements between TM and LiDAR in the healthy volunteer was analyzed using the intraclass correlation coefficient (ICC). The time required for TM and obtaining 3D images by LiDAR was compared using the Wilcoxon signed-rank test. The circumference and volume measurements by LiDAR and TM in a healthy volunteer were compared using Wilcoxon signed-rank test. The Shapiro–Wilk W test was used to verify that the data for the patient group followed a normal distribution. The Pearson correlation coefficient was used to analyze the association between circumference and volume measurements between TM and LiDAR. A paired t-test was used to compare circumference and volume measurements between TM and LiDAR. A Bland–Altman plot was used to analyze the agreement of measurements between TM and LiDAR. The limits of agreement (LOA) were set to mean ± 1.96 SD and plotted. All statistical analyses were performed using JMP Pro® 17 (SAS Institute, Cary, NC, US). Statistical significance was set at *p* < 0.05.

## 3. Results

### 3.1. Evaluation of the Validity and Reliability of LiDAR in a Healthy Volunteer

**Figure 3:**
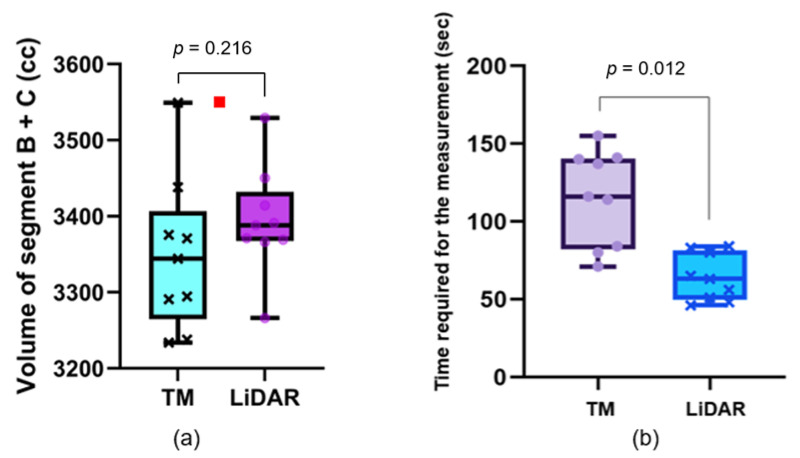
Volume measurements and the time required for each measurement. (**a**) Box and whisker plot of the limb volume in a healthy volunteer. Each plot is obtained from a single measurement. The red square plot shows the volume determined using the water displacement method. (**b**) Box and whisker plots of the time required for each measurement. The time required for measurement by LiDAR is significantly shorter than that required by TM. LiDAR: light detection and ranging, TM: tape measurement.

The healthy volunteer was a 22-year-old male, with a height of 170 cm and a weight of 61 kg. The inter-rater ICCs for the circumference measurements made using TM and LiDAR were 0.998 (95% confidence interval [CI]: 0.995 to 0.999) and 0.988 (95% CI: 0.982 to 0.993), respectively. The inter-rater ICCs for the volume measurements made using TM and LiDAR were 0.954 (95% CI: 0.941 to 0.967) and 0.960 (95% CI: 0.946 to 0.974), respectively. Wilcoxon signed-rank test showed that the time required for the measurements by LiDAR was significantly shorter than that required for TM (64.0 ± 15.1 vs. 115.3 ± 30.6 s, *p* = 0.0012). The average volumes in segments B and C obtained using TM and LiDAR were 3348.3 ± 100.7 and 3393.8 ± 70.8 cc, respectively. The volume determined using the water displacement method was 3550 cc (Figure 3). Table 1 shows the circumference and volume measurements in detail.

### 3.2. Agreement and Correlation Between TM and LiDAR in Patients with LEL

A total of 55 patients participated in this study, and 81 limbs were affected. All patients were female except one. Only two patients had primary lymphedema. The others had secondary lymphedema after cancer treatment, including surgery, chemotherapy, and radiation. The patient characteristics are presented in Table 2.

**Figure 4:**
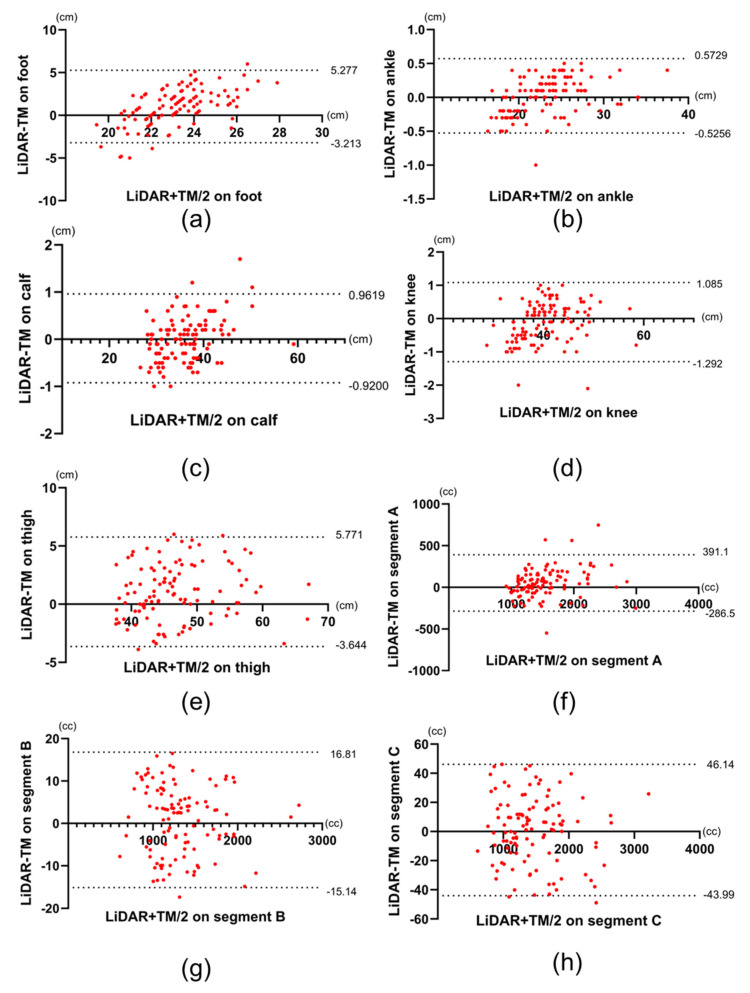
Bland–Altman plots of LiDAR and TM data of all limbs. (**a**–**h**) Bland–Altman plots the measurements obtained from the foot, ankle, calf, knee, thigh, and the volume of segments A, B, and C, respectively. Dotted lines represent upper and lower limits of agreement, respectively. LiDAR: light detection and ranging, TM: tape measurement.

Regardless of the affected or nonaffected limbs, at the foot and thigh, the circumference measurements made using LiDAR were larger than those made using TM (23.7 ± 2.53 vs. 22.7 ± 1.40 cm, *p* < 0.0001 at the foot and 47.4 ± 6.61 cm vs. 46.3 ± 6.31 cm, *p* < 0.0001 at the thigh). Furthermore, the percentage of LOA to the mean was also high at these sites (35.8% for the foot and 19.8% for the thigh; Figure 4 and Table 3). Similarly, in the volume of Segment A in all limbs, the foot and thigh of only affected limbs, the volume made using LiDAR were larger than that made using TM (1575 ± 455 vs. 1523 ± 411 cc, *p* < 0.0001), and the percentage of the differences in LOAs to the mean was also high on the volume of segment A (42.9%).

**Figure 5:**
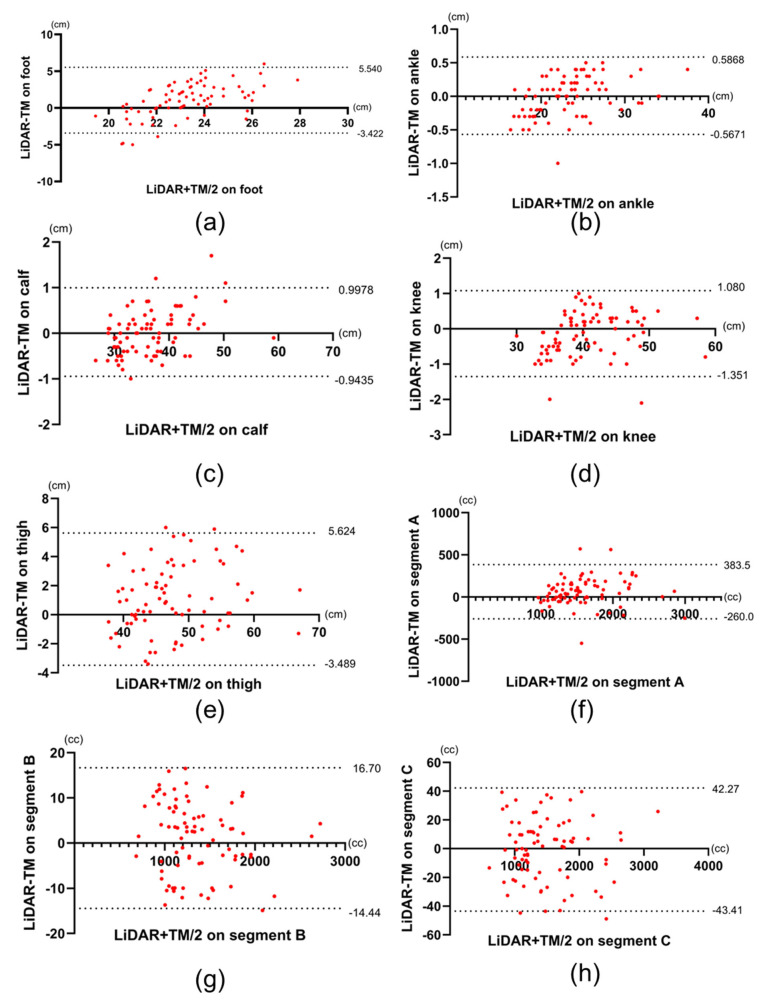
Bland–Altman plots of LiDAR and TM data of the affected limbs. (**a**–**h**) Bland–Altman plots the measurements obtained from the foot, ankle, calf, knee, thigh, and the volume of segments A, B, and C, respectively. Dotted lines represent upper and lower limits of agreement, respectively. LiDAR: light detection and ranging, TM: tape measurement.

In only affected limbs, the circumference measurements at foot and thigh and the volume in segment A made using LiDAR were larger than those made using TM (23.8 ± 2.57 vs. 22.7 ± 1.43 cm, *p* < 0.0001 at the foot, 48.0 ± 6.70 vs. 46.9 ± 6.31 cm, *p* < 0.0001 at the thigh, and 1623 ± 442 vs. 1561 ± 425 cc, *p* < 0.0001 in segment A). In addition, the percentage of the differences in LOAs to the mean was also high in these sites (37.6% for the foot, 18.9% for the thigh, and 39.5% for the segment A; Figure 5 and Table 3). Figure 4 and Figure 5 show the Bland–Altman plots comparing TM with LiDAR measurements on all limbs and only on the affected limbs, respectively. The upper and lower LOAs at each site and the volume, the difference between upper and lower LOAs, and the percentages of difference in LOAs to the mean measurements using LiDAR are presented in Table 3 and Table 4.

## 4. Discussion

In this study, we evaluated the efficacy of lower limb measurements made using LiDAR in patients with LEL by comparing LiDAR measurements with TM. The efficacy of LiDAR in surveying the terrain, including rivers, architectural sites, and forests, has been reported in the literature [9,10,11]. However, only a few studies have demonstrated its effectiveness in the field of biometrics. The present study showed that measurements made using LiDAR were as reliable as TM and that the time required to obtain 3D information for measuring the circumference was shorter with LiDAR than with TM. In patients with LEL, there was no significant difference between LiDAR and TM in terms of circumference measurements at the ankle, calf, and knee, and the volume of segments B and C, regardless of whether the limb was affected or not. the percentages of the differences in LOAs to the mean were low in these sites. This shows that the two measurements were strongly correlated with each other in the ankle, calf, thigh, and segments B and C. However, in the foot, thigh, and segment A, the circumference and volume measurements made using LiDAR were larger than those made using TM, and the percentage of the differences in LOAs to the mean were high. Based on these results, the circumference measurements at the ankle, calf, and knee as well as volume in segment B and C measurements made using LiDAR showed a high agreement with those made using TM, whereas the agreement between the measurements at the foot and thigh as well as volume in segment A was lower.

The ease of use and accuracy of the measuring device are the key factors in performing measurements on the human body [12]. Several methods, including TM, perometry, stereophotogrammetry, and artificial intelligence, have been developed for leg measurements [10,13,14,15]. LiDAR is used in autonomous vehicles for navigation and environment mapping [3,4], and the accuracy of precision instruments equipped with LiDAR is reportedly in the order of a few millimeters [16]. For leg measurements, handheld devices are superior to fixed devices because it is necessary to acquire data from the inseam to obtain point cloud data of the entire lower extremity. Smartphones equipped with LiDAR are small and easy to handle, making them suitable for capturing 3D images of the lower limbs.

According to the manufacturer, the iPhone’s LiDAR system is capable of capturing depth data at up to 30 frames per second [8]. However, recent studies have reported that the effective sampling rate of the LiDAR-derived depth map is approximately 15 Hz [17]. While this relatively low frame rate may pose limitations for capturing rapidly moving objects, it has minimal impact when scanning stationary targets. In our study, the scanned limbs remained static, and the device was moved slowly around the object to ensure accurate data acquisition. Despite the need for controlled scanning speed, the total time required to acquire a 3D image using LiDAR remained shorter than that required for manual tape measurements. The point cloud density obtained by the iPhone LiDAR sensor varies depending on the scanning distance. At a short range of approximately 25 cm, the theoretical point density can reach up to 7225 points per square meter. However, at a distance of 2.5 meters, the density significantly decreases to approximately 150 points per square meter [18]. This distance-dependent attenuation of spatial resolution should be taken into account when designing scanning protocols for clinical or research applications.

In addition, the 3D Scanner App® used in this study allows users to manually configure the point interval, with available settings ranging from approximately 5 to 20 mm [19]. While setting the interval to 5 mm enables extremely high-density data acquisition, it also limits the maximum scannable area due to memory and processing constraints. Therefore, for broader surface scans, resolutions in the range of 10–15 mm are generally recommended. In our study, we employed the 5 mm setting to achieve finer detail in limb geometry, prioritizing resolution over scan extent.

In this study, the percentages of difference in LOAs to the mean measurements made at the ankle, calf, and knee as well as the volume in segments B and C were less than 6.6%. The LOAs shows the measurement error between the two tests; therefore, a small LOAs indicates a high degree of agreement between the two tests [20]. Previous studies showed that the minimal detectable change (MDC) in TM is 6.6% [21]. The MDC is a critical statistical concept used to differentiate true changes from measurement errors. When the difference in LOAs between the two tests is less than the MDC of one of the gold standard tests, the agreement between the two tests is considered high [22]. In the present study, the differences in LOAs between TM and LiDAR measurements made at the ankle, calf, and knee and the volume in segment B and C were smaller than 6.6%, reported as the MDC of TM. This indicates that the agreement was sufficiently high to be considered in the same test. The high ICC between the two tests at the ankle, calf, and knee and the volume in segments B and C in a healthy volunteer also supported this result. 

**Figure 6:**
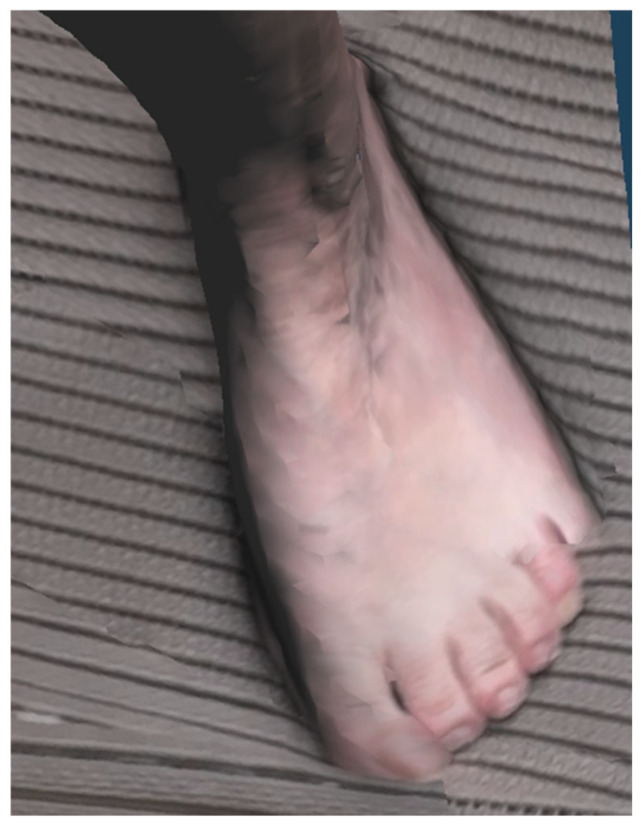
3D images obtained by LiDAR. The acquired image is blurred and the boundary with the floor is unclear. 3D, three-dimensional; LiDAR, light detection and ranging.

The percentage of difference in LOAs to the mean measurements at the foot and thigh was greater than 6.6%. A possible reason for this is that the initially captured data were primarily obtained from the lower leg, excluding the foot and thigh. The foot is located on a plane that differs from the long axis of the lower leg. Therefore, we considered that a measurement error occurred because it is difficult to acquire 3D image data of the foot and lower leg simultaneously. Another possible cause of measurement error is that one plantar surface is in contact with the ground, making it difficult to acquire data (Figure 6). In addition, the developer of the 3D Scanner App® recommends that users move the device slowly and scan larger areas in multiple passes to improve data capture [19]. This approach may improve the accuracy of circumferential measurements in the foot region. However, because our study focused on evaluating the feasibility of quick and simple LiDAR-based circumferential measurements, we intentionally prioritized simplicity over complex scanning maneuvers.

In contrast, measurement errors in the thigh region may arise from different causes. Previous studies showed that the thigh region exhibits greater measurement error and variability when assessed with TM, primarily due to the abundance of soft tissue and the technical difficulty in achieving consistent tape tension and placement [23,24,25]. This may explain why the average values of LiDAR measurements were higher than those of TM in this study. This error may have also affected the volume errors in segment A. It is also possible that the LiDAR measurements were more accurate than TM in this region. However, verifying this would require comparison with a third, independent modality such as 3D optical scanning, which was beyond the scope of the present study.

In the context of LEL management, the clinical relevance of the foot and thigh regions is generally limited. The circumference of the foot is not typically used for the selection or fitting of compression garments. Instead, the ankle serves as the primary reference point due to its anatomical consistency and importance in establishing adequate pressure gradients. Compression garments are then fitted to the calf, knee, and thigh in descending order of clinical priority, and in some cases—particularly in mild or localized edema—garments are prescribed for the lower leg only, without including the thigh [26,27,28]. Moreover, in volumetric assessments using TM, the truncated cone approximation method is widely employed. This technique assumes a cylindrical limb shape and circular cross-sections, which are not applicable to the irregular and non-conical structure of the foot. Therefore, areas distal to the ankle are frequently excluded from volume estimations in both clinical and research settings [29]. Taken together, these anatomical and methodological limitations suggest that the reduced measurement accuracy observed with LiDAR in the foot and thigh regions is unlikely to affect its overall utility in LEL evaluation.

The time required for measurement by LiDAR was significantly shorter than that required for TM. Circumference measurement is one of the most important evaluation methods for LEL because these measurements are needed to create well-fitting compression garments for patients with LEL [1]. However, TM is time-consuming and burdensome for both the patient and the examiner. This study showed that circumferential measurements made using 3D images acquired by LiDAR have the potential to make measurements at arbitrary cross sections easier and faster. This advantage can be maximized with tailor-made garments that have more measurement points but are fitter and more effective for LEL treatment.

The inter-rater ICC of LiDAR measurements was high, despite all examiners having little or no experience in handling LiDAR data. These results suggest that patients and their caregivers may be able to make accurate measurements using LiDAR on their own. The iPhone 15 Pro® used in this study can be purchased by anyone for USD 999, which is another reason why it is relatively patient-friendly.

If patients can easily measure their affected limbs, they will no longer need to visit a doctor to make a garment, thereby reducing the burden on patients living in remote areas. Moreover, the daily status of the affected limb can be digitally archived for use in medical practice. This could benefit patients, as a form of teledermatology that has been under development in recent years [30].

This study has some limitations. First, the manufacturer did not provide detailed specifications for the LiDAR sensor integrated into the device. Therefore, it is unclear whether other LiDAR devices can perform similar measurements [8]. Second, although our healthy volunteer study followed internationally accepted minimum standards for measurement system analysis (MSA), the limited number of samples (n = 5) may restrict generalizability. Increasing the sample size may enhance the statistical power and robustness of reliability estimates, and this should be considered in future studies [31,32]. Finally, the current workflow remains complex. In this study, we used a commercially available app (3D Scanner App®) to capture 3D images, but the measurement analysis was performed separately using an external 3D processing software. In addition, anatomical complexity, particularly in the foot, makes it difficult to acquire complete and accurate scans in a single pass. These issues currently hinder patients from performing independent measurements using a smartphone alone. In the future, developing an integrated application that combines 3D scanning and automated circumference analysis—specialized for lower limb lymphedema—would make LiDAR-based measurement truly patient-friendly. Such tools could support remote monitoring and expand access to care for patients in underserved regions.

## 5. Conclusions

This study showed that a smartphone with LiDAR is a reliable and time-efficient tool for measuring the circumference and volume of the ankle, calf, and knee in patients with LEL. Although improvements are required for the foot and thigh regions, this technology offers substantial potential for reducing patient burden and enhancing personalized garment fitting.

## Figures and Tables

**Table 1:** Circumference and volume measurement in a healthy volunteer.

	LiDAR (n = 9)	TM (n = 9)	*p* Value
Foot (cm)	23.9 ± 1.57	23.8 ± 0.413	0.690
Ankle (cm)	21.3 ± 1.32	21.6 ± 0.371	0.623
Calf (cm)	35.9 ± 0.810	36.0 ± 0.534	0.756
Knee (cm)	37.9 ± 0.995	37.7 ± 0.296	0.626
Thigh (cm)	44.6 ± 0.596	44.7 ± 0.454	0.722
Segment A (cc)	1369.9 ± 30.5	1353.4 ± 20.8	0.216
Segment B (cc)	1826.6 ± 42.8	1783.7 ± 120.7	0.289
Segment C (cc)	1567.2 ± 54.4	1564.7 ± 151.7	0.929

Continuous variables are expressed as means ± standard deviation. LiDAR: light detection and ranging, TM: tape measurement.

**Table 2:** Characteristics of Patients.

		Patients (n = 55)	Affected Limbs (n = 81)
Age (year)		64.1 ± 11.4	-
Causes of lymphedema	Endometrial cancer	28	-
	Ovarian cancer	12	-
	Cervical Cancer	10	-
	Primary lymphedema	2	-
	Others	3	-
BMI		23.8 ± 5.10	-
ISL classification per limb	1	-	55
	2a	-	15
	2b	-	10
	3	-	1
Lymphedema duration(month)		-	56.7 ± 73.6

Continuous variables are expressed as means ± standard deviation. ISL: International Society of Lymphology, BMI: body mass index.

**Table 3:** Average of the Measurements, Correlation Coefficient, and LOAs for all Limbs.

	LiDAR (cm/cc)	TM (cm/cc)	*p* Value	Correlation Coefficient	*p* Value	△LOAs (cm/cc)	%△LOAs to the Mean Measurement Made Using LiDAR
Foot	23.7 ± 2.53	22.7 ± 1.40	<0.0001	0.519	<0.0001	8.49	35.8
Ankle	23.2 ± 4.10	23.1 ± 3.98	0.378	0.998	<0.0001	1.09	4.70
Calf	36.2 ± 5.77	36.2 ± 5.58	0.648	0.997	<0.0001	1.88	5.19
Knee	40.3 ± 5.48	40.4 ± 5.32	0.758	0.994	<0.0001	2.38	5.90
Thigh	47.4 ± 6.61	46.3 ± 6.31	<0.0001	0.932	<0.0001	9.41	19.8
Segment A	1575 ± 455	1523 ± 411	0.0020	0.925	<0.0001	677	42.9
Segment B	1327 ± 372	1326 ± 373	0.285	0.999	<0.0001	31.9	2.40
Segment C	1461 ± 480	1460 ± 483	0.625	0.998	<0.0001	90.0	6.16

Continuous variables are expressed as mean ± standard deviation. △LOAs: Difference in the limits of agreement, LiDAR: light detection and ranging, TM: tape measurement.

**Table 4:** Average of the Measurements, Correlation Coefficient, and LOAs in the Affected Limbs.

	LiDAR (cm/cc)	TM (cm/cc)	*p* Value	Correlation Coefficient	*p* Value	△LOAs (cm/cc)	%△LOAs to the Mean Measurement Made Using LiDAR
Foot	23.8 ± 2.57	22.7 ± 1.43	<0.0001	0.466	<0.0001	8.96	37.6
Ankle	23.4 ± 4.36	23.3 ± 4.24	0.763	0.998	<0.0001	1.14	4.87
Calf	36.6 ± 6.10	36.5 ± 5.87	0.623	0.997	<0.0001	1.93	5.27
Knee	40.7 ± 5.64	40.8 ± 5.50	0.0522	0.994	<0.0001	2.43	5.97
Thigh	48.0 ± 6.70	46.9 ± 6.31	<0.0001	0.937	<0.0001	9.10	18.9
Segment A	1623 ± 442	1561 ± 425	0.0011	0.929	<0.0001	642	39.5
Segment B	1351 ± 393	1349 ± 394	0.204	0.998	<0.0001	31.3	2.31
Segment C	1485 ± 514	1486 ± 516	0.815	0.999	<0.0001	85.6	5.76

Continuous variables are expressed as means ± standard deviation. ICC: intraclass correlation coefficient, △LOAs: difference of limits of agreement, LiDAR: light detection and ranging, TM: tape measurement.

## Data Availability

The raw data supporting the conclusions of this article will be made available by the authors on request.

## Abbreviations

The following abbreviations are used in this manuscript:

TM	Tape measurement
LiDAR	Light detection and ranging
LOA	Limits of agreement
3D	Three-dimensional
BMI	Body mass index
ISL	International Society of Lymphology
LEL	Lower extremity lymphedema
ICC	Intraclass correlation coefficient
CI	Confidence interval
